# Synthesis of NiRu-Layered Double Hydroxide for Enhanced Oxygen Evolution Reaction

**DOI:** 10.1021/acsomega.5c12581

**Published:** 2026-01-31

**Authors:** Eden Argaw, Binod Raj KC, Bishnu Prasad Bastakoti

**Affiliations:** Department of Chemistry, North Carolina A&T State University, 1061 E. Market St, Greensboro, North Carolina 27411, United States

## Abstract

Nickel–ruthenium layered double hydroxide (NiRu-LDH) was synthesized by coprecipitation using formamide as both solvent and intercalant. The synthesis was performed at various temperatures to study the impact of temperature on electrochemical performance. Temperature plays a vital role in optimizing LDH synthesis to enhance oxygen evolution reaction (OER) efficiency. The optimal temperature yielded a well-defined morphology with balanced crystallinity, leading to more active sites and improved charge transfer. Electrochemical tests indicated that the NiRu-LDH sample prepared at 50 °C (NiRu-LDH-50) required only 280 mV overpotential versus the reversible hydrogen electrode to achieve 10 mA/cm^2^ and exhibited a low Tafel slope of 52 mV/dec, demonstrating excellent catalytic kinetics. This study presents the synthesis of NiRu-LDH via coprecipitation and emphasizes how reaction temperature influences LDH structural properties and OER performance.

## Graphical abstract

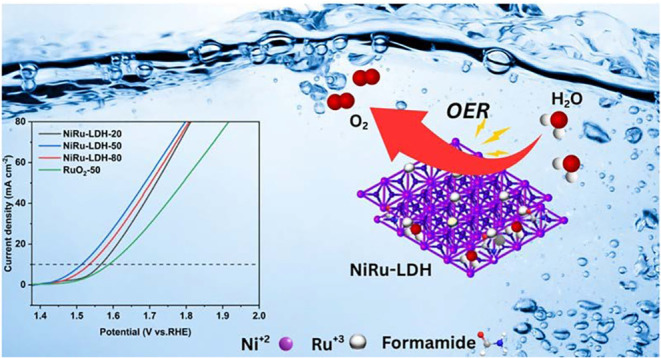

## 1 Introduction

Water splitting is a highly sustainable approach to clean energy production, enabling the direct generation of hydrogen and oxygen from water using renewable electricity.^1–4^ The process comprises the hydrogen evolution reaction (HER) at the cathode and the oxygen evolution reaction (OER) at the anode, with water electrolysis being widely recognized as the cleanest and most promising method for large-scale hydrogen production.^5^ However, the sluggish reaction kinetics and the resulting high overpotentials associated with both the OER and HER represent major obstacles to generating hydrogen through water splitting.^6,7^ Recent theoretical and experimental studies have demonstrated that ruthenium exhibits outstanding adsorption properties for key reaction intermediates in OER, making it a highly effective component for boosting catalytic activity.^8^ The theoretical minimum energy required for water splitting is Δ*G* = 237.1 kJ mol^–1^ at a thermodynamic potential of 1.23 V vs RHE.^9^ However, due to the kinetic constraints of OER, a potential higher than the thermodynamic value is typically required to drive the process.^1,9^ This excess potential is referred to as overpotential (η), which reflects the inefficiency associated with the reaction’s sluggish kinetics.^10^ A large overpotential indicates a low energy conversion efficiency during electrochemical water splitting. This means that more electrical energy is required to drive the reaction, thereby reducing the overall energy conversion efficiency in water electrolysis.^11^ In addition to overpotential, the Tafel slope and exchange current density can also be used to evaluate the catalyst’s performance from the overpotential–kinetic current relationship. A lower Tafel slope indicates a greater increase in current density with changes in overpotential, reflecting faster electrocatalytic reaction kinetics.^12^ Conversely, a higher exchange current density at the catalyst surface suggests enhanced catalytic activity, indicating a reduced reaction barrier and an accelerated charge-transfer rate.^13^

Among various electrocatalysts, layered double hydroxides (LDHs) are widely used for OER due to their simplicity, cost-effectiveness, scalability, and interlayer spacing. At present, nickel-based LDH structured catalysts are widely recognized as the most effective OER catalysts under alkaline conditions.^14^ To further enhance their OER performance, Ni-based LDHs have been modified by incorporating higher-oxidation-state transition metals, such as Ir and Ru, thereby representing a significant advancement in catalyst design.^15,16^ Scientists have increasingly focused on integrating noble metals such as Ru with transition metals like Ni to overcome the inherent kinetic limitations of the OER and to modulate electronic structures for enhanced catalytic performance.^17,18^ Ru-based catalysts are desirable because it offers high intrinsic activity, favorable adsorption energies for OER intermediates, and enables reduced overpotentials at benchmark current densities (10 mA cm^–2^) compared with many first-row transition metal oxides.^19^ However, these catalysts often suffer from poor long-term stability and structural reconstruction under operating conditions, and existing synthesis approaches frequently involve complex procedures or high temperatures that are not energy-efficient or scalable.^20^ Consequently, recent research has turned toward rational synthesis optimization, including controlled dopant coordination, defect engineering, and temperature-regulated methods to fine-tune the catalyst structure and active site distribution while balancing activity, stability, and energy input requirements.^21^ This perspective motivates the present work, which systematically investigates the effect of synthesis temperature on the structure activity relationships of NiRu-LDH catalysts for alkaline OER.

Reaction temperature during the synthesis of LDHs is a critical factor that significantly influences their catalytic performance in OER.^22^ Temperature affects the key structural and morphological properties of LDHs, including crystallinity, layer stacking, surface area, and metal ion distribution.^23^ These characteristics directly impact the availability of active sites and the efficiency of charge transfer during OER.^24^ Careful control and study of these parameters enable the development of more efficient and stable LDH-based electrocatalysts. Optimizing temperature within the appropriate range for the chosen synthesis method enables the design of LDH catalysts, thereby improving electrocatalytic performance. The findings highlight the impact of reaction temperature on the performance of NiRu-LDH and its potential as an efficient catalyst for OER applications.^25^

In this study, NiRu-LDH was synthesized via coprecipitation at varying reaction temperatures to investigate the effect of synthesis temperature on OER performance. Cyclic voltammetry (CV) curves displayed well-defined redox peaks, indicating robust electrochemical activity. The high current densities observed suggest a large electrochemically active surface area and efficient charge transfer within the catalyst network. The overpotential required to reach a current density of 10 mA cm^–2^, as observed from the linear sweep voltammetry (LSV) curve, was 280 mV for the NiRu-LDH-50 sample, which is comparable to many values reported in the literature. This superior performance can be attributed to the optimized reaction temperature, which promotes a well-defined nanostructure, balanced crystallinity, and uniform morphology, thereby exposing more active sites and enhancing charge transfer. In contrast, NiRu-LDH synthesized at 20 °C temperature (NiRu-LDH-20) shows incomplete structural development, while the 80 °C temperature sample (NiRu-LDH-80) suffers from particle agglomeration, both of which decrease the number of active sites and hinder electron mobility, thereby confirming 50 °C as the most favorable synthesis condition for efficient OER. By systematically correlating synthesis temperature with structural evolution and OER performance, this work provides new insights into optimized energy synthesis strategies and structure–activity relationships in NiRu-LDH catalysts.

## 2 Experimental Section

### 2.1 Materials

Ruthenium­(III) chloride (RuCl_3_, 99.9%, anhydrous, trace metal basis) and nickel chloride hexahydrate (NiCl_2_·6H_2_O, ≥98%) were purchased from Thermo Scientific. Sodium hydroxide (NaOH, ≥97%), potassium hydroxide (KOH, 90%), and poly­(vinylidene fluoride) (PVDF) were purchased from Sigma-Aldrich. 1-Methyl-2-pyrrolidinone (NMP, 99.5%) and carbon black, acetylene (≥99.9%, 50% compressed) were purchased from Alfa Aesar. Formamide (CH_3_NO) was purchased from Fisher Chemicals. Carbon cloth was obtained from Fuel Cell Earth. All chemicals were used as received without further purification, and distilled water was used in all experiments.

### 2.2 Synthesis of Ni–Ru LDH

Ni–Ru LDHs were prepared by coprecipitation (Figure 1a). NiCl_2_·6H_2_O and RuCl_3_ were used in a 3:1 ratio and dissolved in 10 mL of distilled water. A formamide solution was prepared by adding 4 mL of formamide to 16 mL of distilled water, serving as an anion source and interlayer spacing agent that facilitates exfoliation into few-layer nanosheets. The salt solution was added dropwise to the formamide solution, and 0.5 M NaOH was added simultaneously to maintain a pH of 9–10. The reaction was carried out at temperatures of 20, 50, and 80 °C, with each sample stirred for 2 h. The samples were labeled NiRu-LDH-20, NiRu-LDH-50, and NiRu-LDH-80. The numbers correspond to the reaction temperatures in °C. After cooling to room temperature, the product was collected by centrifugation, washed several times with ethanol and distilled water, and then dried in a vacuum oven at 60 °C for 12 h. RuO_2_ is also prepared using the same method at an optimal temperature of 50 °C, without Ni^2+^ ions labeled as RuO_2_-50, for further investigation and comparison with other samples.

**Figure 1:**
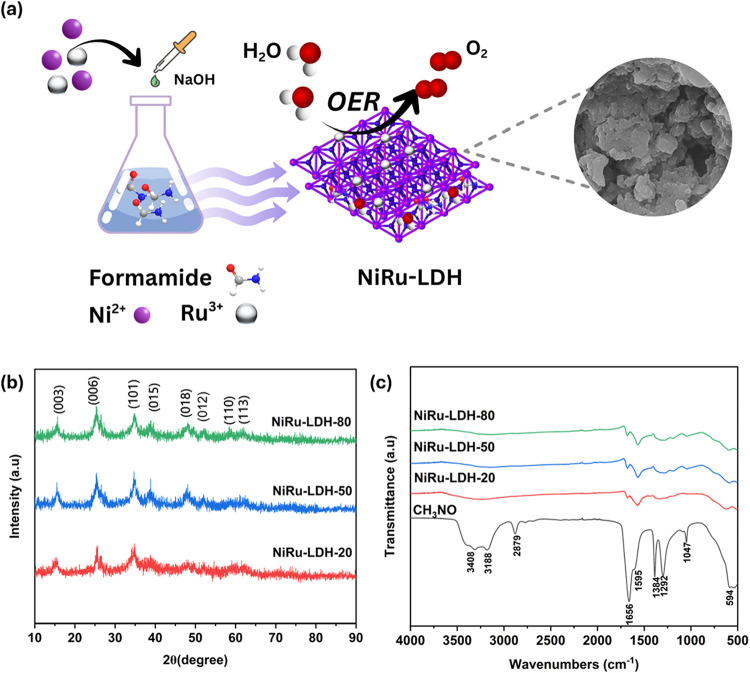
(a) Schematic illustration of the synthesis procedure of NiRu-LDH and structural coordination of NiRu-LDH, (b) XRD spectra of NiRu-LDH with different reaction temperatures, (c) Comparison of FTIR spectra of formamide and NiRu-LDH with varying temperatures of reaction.

### 2.3 Preparation of the Electrode

The prepared materials were used as working electrodes for electrochemical tests. First, 5.0 mg of the catalyst was finely ground and mixed with 500 μL of NMP. Then, 1.0 mg of carbon black and 1.0 mg of PVDF binder were added, and the mixture was sonicated for 1 h to ensure even dispersion. Small strips of carbon cloth (1.0 cm × 1.0 cm) were first rinsed with distilled water and then immersed in a 100 mL solution of concentrated nitric and sulfuric acids (1:3 v/v). The solution was heated at 60 °C for 2 h. Once cooled to room temperature, the cloth was thoroughly washed with distilled water and dried at 60 °C for 6 h. After that, 100 μL of the prepared electrode suspension was drop-cast onto the treated carbon cloth, and the resulting electrode suspension was further dried in a vacuum oven at 60 °C for 12 h. Electrochemical measurements were performed using a Biologic VMP3 instrument. An Ag/AgCl electrode served as the reference electrode, a platinum wire as the counter electrode, pretreated carbon cloth as the substrate, and the synthesized materials as the working electrode in 1 M KOH solution. The electrode potentials were converted to the reversible hydrogen electrode (RHE) scale using the equation: *E* (vs RHE) = *E* (Ag/AgCl) + 0.198 + 0.059 × pH.^9^

### 2.4 Characterization

The surface morphology of the synthesized samples was analyzed using field emission scanning electron microscopy (FESEM, JEOL, JSM-IT800). The internal structure, crystallinity, and chemical composition were investigated using high-resolution transmission electron microscopy (HR-TEM) operated at an accelerating voltage of 200 kV from JEOL JEM-2100PLUS with a STEM/EDX capability. To analyze the elemental distribution and chemical composition of the catalyst, energy-dispersive X-ray spectroscopy (EDX) and elemental mapping were performed using an Oxford Instruments system. X-ray diffraction (XRD) patterns were recorded using a Rigaku MiniFlex 600 diffractometer equipped with a scintillation counter detector and a Cu Kα radiation source. The measurements were conducted at 40 kV and 15 mA to confirm the crystalline phases of NiRu-LDH. Bragg’s law was employed to determine the *d*-spacing and c-lattice parameters of the synthesized material. This method allows precise calculation of interplanar distances from XRD data. The average crystallite size (*D*) was calculated using the Scherrer equation

1 $$D=\frac{K\lambda }{\beta \cos\theta }$$

where *K* is the shape factor (0.9), λ is the X-ray wavelength (1.5406 Å), β is the full width at half-maximum (FWHM) of the diffraction peak, and θ is the Bragg angle.^26^ Furthermore, the functional groups present in the synthesized material were identified by Fourier transform infrared (FTIR) spectroscopy using the IRTracer-100 instrument. X-ray photoelectron spectroscopy (XPS) analysis was performed using a Thermo Scientific ESCALAB XI system with an Al Kα source and 200 eV energy to determine the chemical composition and oxidation states of elements present in the samples.

## 3 Results and Discussion

### 3.1 Structural and Morphological Characterizations

Figure 1b shows the diffraction peak of NiRu-LDH synthesized at different temperatures. The main NiRu-LDH peak positions are 15.1°, 25.5°, 33.4°, and 60°, corresponding to the (003), (006), (012), and (110) planes.^27^ The (003) basal plane in LDHs is the primary XRD indicator of interlayer spacing, which varies with the type of intercalated anions and water content.^28^ The synthesized NiRu-LDH shows similar diffraction patterns at all reaction temperatures; however, the peak intensity and crystallinity of LDH increase with higher temperatures than room temperature. Crystallite sizes calculated from the (003) plane using the Scherrer equation (eq 1) were 3.35 nm (NiRu-LDH-20), 7.35 nm (NiRu-LDH-50), and 5.24 nm (NiRu-LDH-80). The smaller size at 20 °C results from insufficient atomic mobility leading to incomplete crystal growth, while the reduced size at 80 °C might be due to structural defects. The optimal size and crystallinity at 50 °C enhance charge transfer and active site exposure, thereby improving OER performance. FTIR spectra, as shown in Figure 1c, were used to confirm the presence of interlayer anions, hydroxyl groups, and the functional groups in the LDH structures. The absorption bands in the range of 1500–1580 and 1350–1450 cm^–1^, corresponding to N–H bending and C–N stretching vibrations, respectively.^29^ These features confirm the presence of formamide in the sample. The broad band around 3400 cm^–1^ is an indication of the stretching mode of the O–H bond in the hydroxide layer.^30,15^ The band between 650 and 500 cm^–1^ is attributed to the vibration stretching modes of O–M–O, M–O–M, and M–O, where M represents either Ru or N. To improve spectral clarity, the FTIR spectrum of the representative NiRu-LDH-50 sample is presented in the Supporting Information Figure S6.

The morphology of the synthesized NiRu-LDH was first examined by SEM (Figure 2a), which revealed a stacked, plate-like architecture typical of LDH structures, providing a high surface area that is beneficial for catalytic activity. As shown in Figure S1, NiRu-LDH-20 exhibits incomplete morphological development due to insufficient crystallization, whereas NiRu-LDH-80 displays particle agglomeration at higher temperatures, confirming that 50 °C provides the most uniform and well-defined morphology. TEM images (Figure 2b,c) further confirm the nanosheet-like morphology, consistent with the layered structure of the LDH material. The SAED pattern (inset of Figure 2c) displays well-defined diffraction rings, confirming the polycrystalline nature of the NiRu-LDH nanocomposite and the diffraction rings/spots that can be indexed to the characteristic LDH planes, with the corresponding plane assignments provided in Figure S3 of the Supporting Information. The annular dark-field scanning transmission electron microscopy (ADF-STEM) image (Figure 2g) and the corresponding EDX elemental maps (Figure 2h–j) of Ni, O, and Ru indicate that these elements are homogeneously distributed throughout the nanosheets, confirming uniform Ru incorporation within the LDH framework. The lattice fringe spacing measured from the HRTEM image corresponds to the (003) plane of NiRu-LDH-50, with an interlayer distance of ∼0.6 nm (Figure S2), consistent with the layered double hydroxide structure.

**Figure 2:**
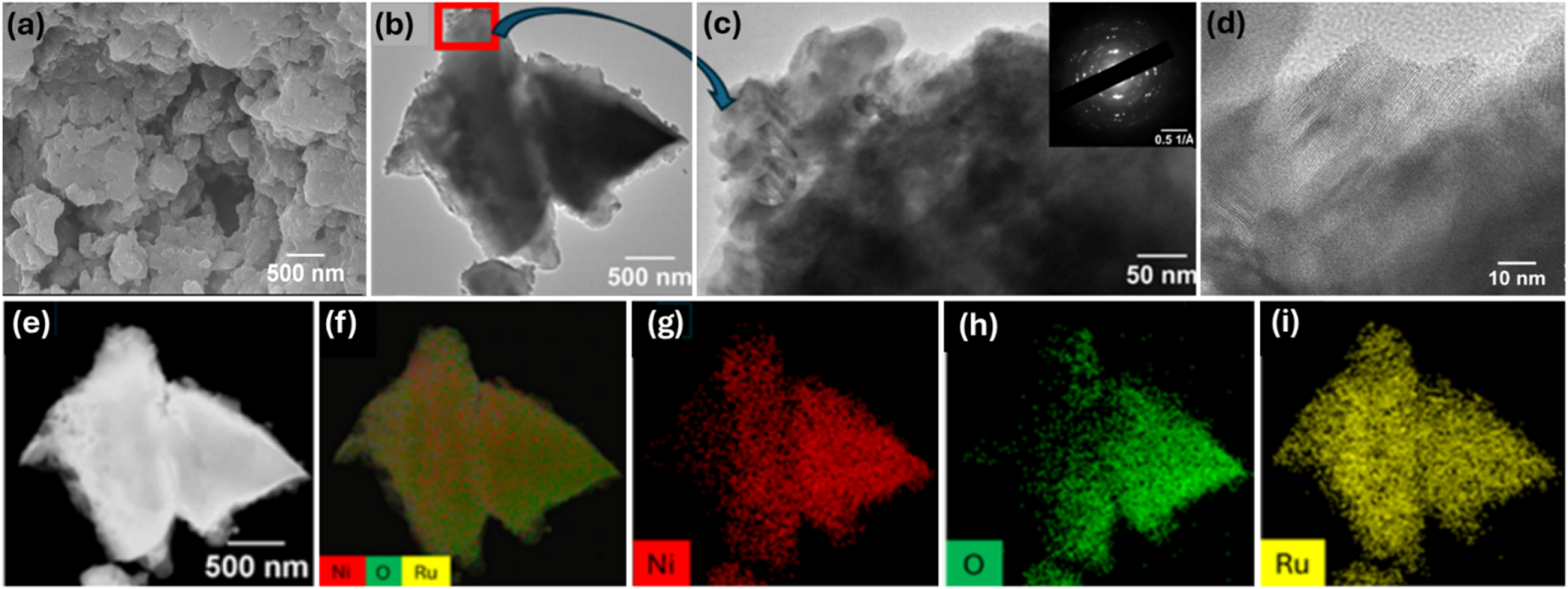
(a) SEM image of NiRu-LDH-50; (b–d) TEM images with inset SAED pattern, (e) scanning transmission electron microscope image, (f) EDX overlay elemental mapping, (g–i) elemental mapping of Ni, O, and Ru.

X-ray photoelectron spectroscopy (XPS) was performed to analyze the surface chemical composition and elemental states of NiRu-LDH. XPS analysis provides insight into the oxidation state and coordination environment of Ru in the NiRu-LDH framework. As shown in Figure 3c, the Ru 3p spectrum displays two main peaks at binding energies of 463.0 eV (Ru 3p_3/2_) and 485.6 eV (Ru 3p_1/2_), which are consistent with Ru^3+^ species reported for Ru-based hydroxides and LDH systems. It is noted that the Ru 3p region may partially overlap with satellite features and contributions from higher oxidation states. Although minor Ru^4+^ contributions cannot be excluded entirely, no distinct additional peaks or shoulders characteristic of Ru^4+^ were clearly resolved within the experimental resolution. Therefore, the Ru species are primarily assigned as Ru^3+^ based on the dominant peak positions.^31,32^ Based on standard binding energies for Ni­(II), the high-resolution Ni 2p spectrum of NiRu-LDH-50 (Figure 3b) displays doublet peaks at 855.6 and 873.1 eV, which are assigned to Ni­(II) species.^33,34^ The additional peaks at 856.8 and 874.5 eV confirm the presence of Ni­(III) species, consistent with reported literature values.^34,35^ Together with the Ni­(II) signals, this demonstrates the coexistence of multiple nickel oxidation states within the NiRu-LDH-50. Such mixed-valence states of Ni, along with the incorporation of Ru, are expected to facilitate charge transfer and provide abundant active sites, thereby significantly enhancing the OER catalytic performance of NiRu-LDH-50. The O 1s XPS spectrum (Figure 3a) exhibits three distinct peaks at approximately 528.7, 530.9, and 532.4 eV, corresponding to Ni/Ru–O (O–III), OH– (O–II), and H–O–H­(O–I) bonds, respectively.^36^ These results confirm the successful formation of both Ni–O and Ru–O chemical linkages within the NiRu-LDH structure. Compared to pristine Ni­(OH)_2_ (Figure 3d), the incorporation of Ru into the LDH framework leads to the emergence of higher-valence Ni^3+^ species, as evidenced by an increased Ni^3+^/Ni^2+^ contribution and a slight positive binding-energy shift in the Ni 2p spectra (Figure 3b), indicating electronic modulation of the Ni centers. After OER testing (Figure 3f), the Ni^3+^-related features become more pronounced, suggesting the stabilization of higher valence Ni species under electrochemical oxidation conditions.^37^ The enhanced Ni^3+^-related features suggest the stabilization of higher valence Ni species under anodic conditions, which is widely considered indicative of the formation of Ni-OOH-like surface species during alkaline OER.^38^ XPS-derived surface atomic percentages, as shown in Table S2, Ru content of around five atomic percentages, confirming effective Ru incorporation into the NiRu-LDH surface.

**Figure 3:**
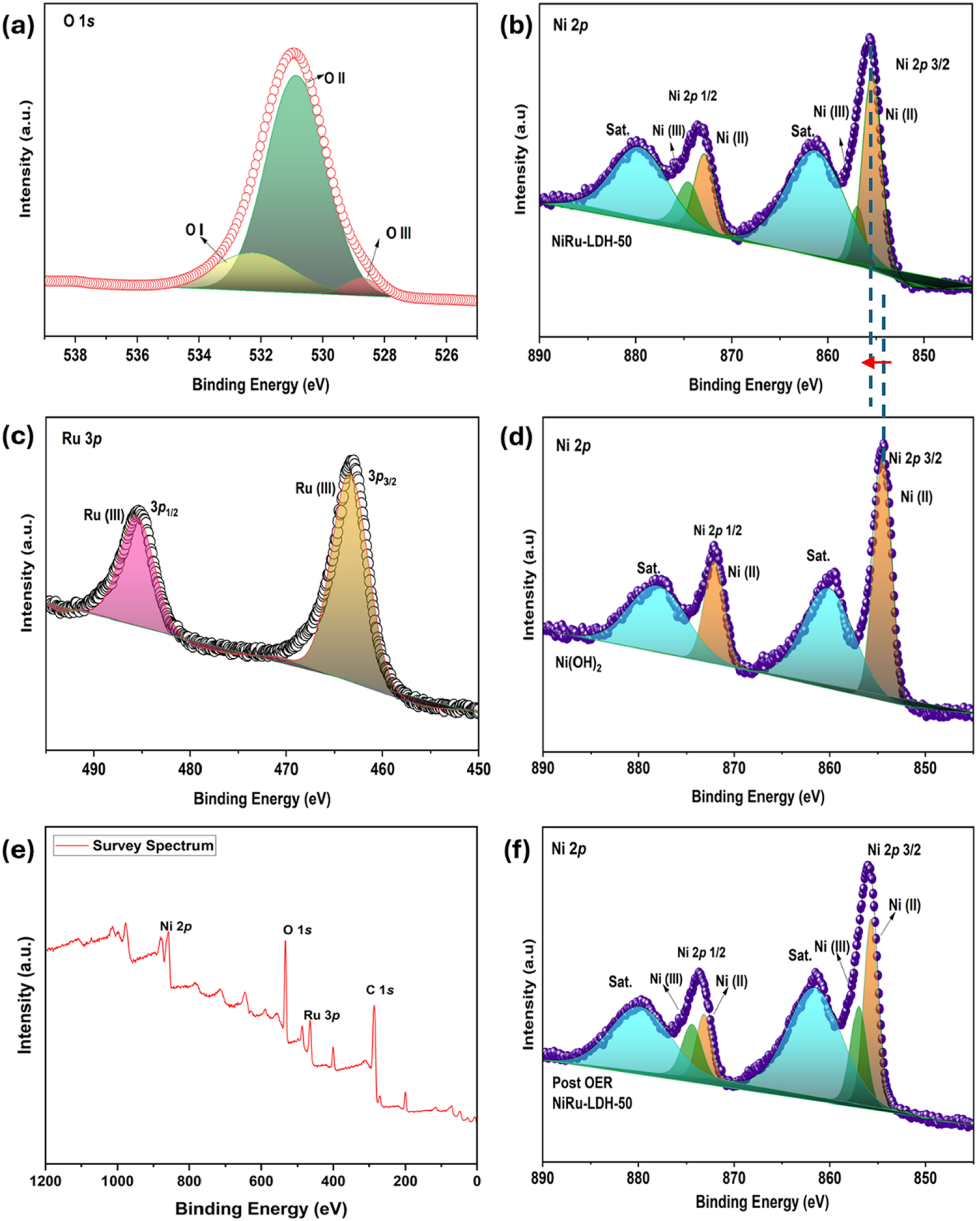
XPS spectra of (a) O 1s for NiRu-LDH-50, (b) Ni 2p for NiRu-LDH-50, (c) Ru 3p for NiRu-LDH-50, (d) Ni 2p for Ni­(OH)_2_, (e) survey spectrum of NiRu-LDH-50, and (f) Ni 2p spectrum of NiRu-LDH-50 after OER.

### 3.2 OER Performance of NiRu-LDH

OER performance of different temperature NiRu-LDHs was investigated in 1 M KOH electrolytes within a potential window of 0.0 to 0.5 V (Vs Ag/AgCl) for CV analysis. The onset potential was 1.51, 1.45, 1.48, and 1.52 V (vs RHE) for NiRu-LDH-20, NiRu-LDH-50, NiRu-LDH-80 and RuO_2_-50_,_ respectively. Lower onset potential and uniform distribution of catalytic performance are crucial for catalytic electrodes.^39,27^ NiRu-LDH-50 exhibits the earliest onset potential and the lowest overpotential to reach 10 mA cm^–2^. The area under the CV curve (Figure S2) and the current response increased with higher scan rates, indicating faster reaction kinetics. Notably, the shape of the CV curve remained consistent across different scan rates, showing the catalyst’s stability and excellent electrochemical performance.^9^ The CV curves of all samples exhibited well-defined redox peaks.

Attributed to the Ni^2+^/Ni^3+^ transition; among them, NiRu-LDH-50 showed a higher anodic peak current density, which, together with its lower charge-transfer resistance observed in EIS, provides clear evidence of better electrochemical activity and faster charge transfer.^40^ Linear sweep voltammetry (LSV) was conducted at a scan rate of 10 mV/s to evaluate the OER activity of the various samples, as illustrated in Figure 4a. LSV analysis revealed that NiRu-LDH-50 achieved the lowest overpotential of 280 mV (Vs RHE) at 10 mA cm^–2^, compared to 300 mV for NiRu-LDH-80, 330 mV for NiRu-LDH-20, and 360 mV for RuO_2_-50 (Figure 4b). The *iR*-corrected LSV curves are provided in the Supporting Information (Figure S10), and the overpotential for NiRu-LDH-50 was reduced to 240 mV after correction. Furthermore, the Tafel slope is a key indicator of the reaction kinetics of electrocatalysts. For NiRu-LDH-20, NiRu-LDH-50, NiRu-LDH-80, and RuO_2_-50 the measured OER Tafel slopes are 126, 52, 60, and 163 mV dec^–1^, respectively, as shown in (Figure 4c). The balanced crystallinity and uniform nanosheets provide more accessible active sites and efficient charge transport, thereby minimizing energy losses during the reaction. This is evidenced by the low Tafel slope of NiRu-LDH-50, lower than that of the other samples, confirming its superior reaction kinetics.^41,10^ As shown in Table S4, NiRu-LDH-50 exhibits an overpotential that is comparable to those of reported NiRu-based and other OER catalysts tested in alkaline media. The stability of a catalyst is a crucial factor in assessing the overall catalytic performance of the synthesized materials, particularly in ensuring their suitability for practical, real-world applications.^42^ The electrochemical stability of the NiRu-LDH-50 catalyst was evaluated using chronoamperometry and CV under alkaline OER conditions. A chronoamperometric test conducted at a constant potential of 0.35 V (vs Ag/AgCl) over 24 h demonstrated a stable current density of approximately 4.5 mA cm^–2^, indicating excellent long-term catalytic stability.^43^ Furthermore, CV cycling was also performed at a scan rate of 50 mV s^–1^ for 1000 continuous cycles (Figure S5). The slight change in the CV curves before and after cycling indicates that the NiRu-LDH-50 catalyst remained stable in both structure and performance. This high durability is attributed to the synergistic interaction between Ni and Ru within the layered double hydroxide matrix, which enhances both electronic conductivity and active site stability.^44^ Electrochemical impedance spectroscopy (EIS) measurements were performed using Z-fit analysis over a frequency range of 100 kHz to 10 mHz in 1 M KOH to evaluate the charge-transfer characteristics of the catalysts (Figure S11). The experimental data revealed that NiRu-LDH-50 exhibited the lowest charge-transfer resistance of 1.16 Ω, indicating superior conductivity and ion diffusion compared to the other samples. NiRu-LDH-20 and NiRu-LDH-80 samples showed increased resistance. RuO_2_-50, used as a reference, displayed the highest resistance among the tested catalysts, further confirming that NiRu-LDH-50 facilitates more efficient charge transfer and enhanced ion diffusion, contributing to its superior electrochemical performance.^45,46^ The equivalent circuit of EIS is presented in Table S1, and the corresponding fitted Nyquist plots are provided in Figure S12. The TOF values of the NiRu-LDH samples were 0.16, 0.2, and 0.12 s^–1^ for the 20, 50, and 80 °C samples, respectively. Calculated at an overpotential of 300 mV using the *iR*-corrected LSV and the equation provided in the Supporting Information (eq S2). Although the LSV of NiRu-LDH-80 shows a higher current density than NiRu-LDH-20, its lower TOF indicates reduced intrinsic activity per site, likely due to particle agglomeration that limits the accessible active sites. In contrast, NiRu-LDH-50 exhibits both high current density and the highest TOF, indicating that the optimized synthesis temperature promotes well-dispersed nanosheets with maximized active site utilization and favorable catalytic kinetics.^47^

**Figure 4:**
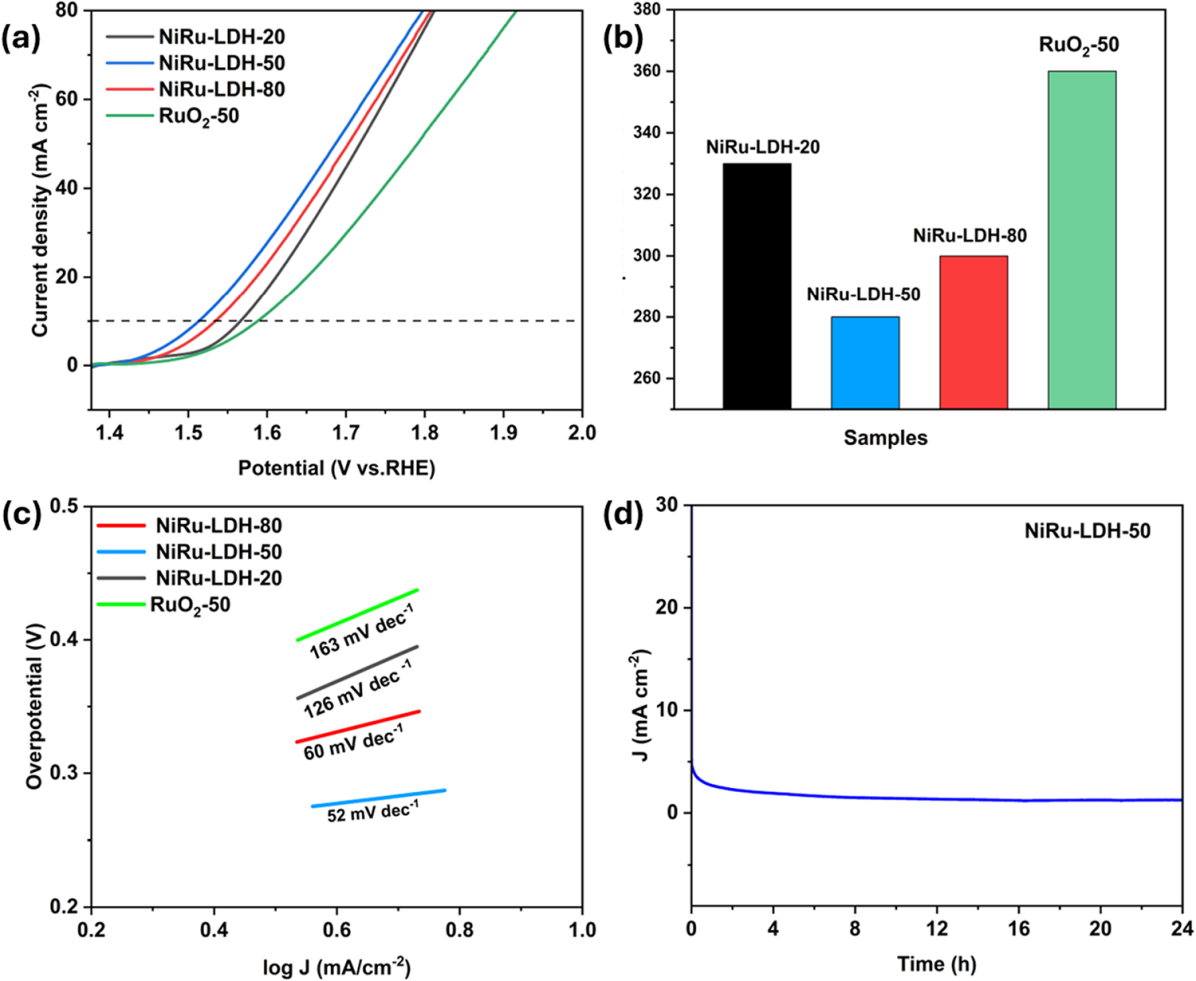
(a) LSV curves of OER activity for different catalysts evaluated in 1 M KOH, (b) graphical representation of the overpotential of different samples at 10 mA cm^–2^, (c) Tafel plot of different samples, and (d) chronoamperometric curve of NiRu-LDH-50.

Before OER, all NiRu-LDH samples exhibit a dominant Raman band around 100 cm^–1^ as shown in (Figure S7), which is attributed to low-frequency lattice vibrations involving O–M–O deformation modes of the layered hydroxide framework.^48^ Additional bands observed around 380 and 540 cm^–1^ are assigned to Ni–O and mixed metal–oxygen (M–O) vibrational modes, respectively, while the band around 680 cm^–1^ is associated with Ru–O vibrations, confirming the successful incorporation of Ru species within the LDH structure.^49,50^ The Raman band observed around 1060 cm^–1^, commonly associated with interlayer organic species, is attributed to C–N stretching vibrations of interlayer formamide.^51^ Its intensity is strongly dependent on the synthesis temperature and reaches a maximum for NiRu-LDH-50. Notably, NiRu-LDH-50 displays markedly higher Raman intensities across all characteristic bands, reflecting enhanced lattice ordering, stronger metal–oxygen coordination, and more effective interlayer stabilization compared to the samples synthesized at 20 and 80 °C.^52,53^ Post-OER ex situ Raman spectra collected after 24 h of chronoamperometry (Figure S7) show that the low-wavenumber lattice vibration around 100 cm^–1^ is preserved, albeit with reduced intensity, indicating retention of the LDH framework. After OER testing the enhanced Ni^3+^ related features, together with the emergence of Raman bands around 380, 480, and 610 cm^–1^, are consistent with the electrochemical oxidation of Ni sites and indicative of modified Ni–O vibrational modes, suggesting surface oxidation of Ni centers during OER, in agreement with literature reports on electrochemically evolved Ni-based hydroxide catalysts.^54^ These changes suggest partial surface reconstruction of the metal–oxygen network during OER, while maintaining the overall structural integrity of the NiRu-LDH catalyst.^55^ Highly intense peaks emerge in the 1200–1400 and 1500–1700 cm^–1^ regions are from the carbon cloth.^56^

The electrochemically active surface area (ECSA) of the NiRu-LDH catalysts was estimated from the double-layer capacitance (*C*_dl_) eq (S1) and the ECSA equation eq (S2), obtained by cyclic voltammetry in a nonfaradaic potential region (Figure S9). The calculated Cdl and corresponding ECSA values for all samples are summarized in Table S3 in the Supporting Information. The NiRu-LDH-50 sample exhibits the highest *C*_dl_ value (12.57 mF cm^–2^), corresponding to an ECSA of approximately 314 cm^2^, which is significantly larger than those of NiRu-LDH-80 (9.47 mF cm^–2^, 237 cm^2^) and NiRu-LDH-20 (5.05 mF cm^–2^, 126 cm^2^). The enhanced ECSA of NiRu-LDH-50 indicates a greater number of electrochemically accessible active sites, which can be attributed to its optimized nanosheet morphology and balanced crystallinity.^57,58^ This enlarged active surface area is consistent with the superior OER activity observed for NiRu-LDH-50, as evidenced by its lower overpotential and smaller Tafel slope.

## 4 Conclusions

In this study, a NiRu-LDH electrocatalyst was successfully synthesized via coprecipitation at varying reaction temperatures to optimize its OER performance. Comprehensive electrochemical characterizations confirmed the formation of nanosheet morphology and uniform elemental distribution. The catalyst synthesized at 50 °C (NiRu-LDH-50) demonstrated superior electrocatalytic activity, with a lowest overpotential of 280 mV at 10 mA cm^–2^ and a high current density (>50 mA cm^–2^), indicating excellent conductivity and abundant active sites for OER. The simplicity of the coprecipitation method used in this study makes it highly suitable for large-scale production of NiRu-LDH catalysts. This approach does not require complex procedures, thereby facilitating scalability. Additionally, optimizing the reaction temperature is crucial because it directly affects the catalyst’s electrochemical performance.

## Supplementary Material



## Notes

SEM images, TEM images, XRD, Raman spectra, FTIR, CV, LSV, EIS, *C*_dl_, ECSA table, Nyquist plot, comparison table (PDF)
