# Supplementary material for: Synthesis of NiRu-Layered Double Hydroxide for Enhanced Oxygen Evolution Reaction
Source: ACS Omega. 2026 Jan 31;11(6):10674–82. doi: 10.1021/acsomega.5c12581 (PMC12917784; doi:10.1021/acsomega.5c12581)
Supplement: Supplementary file 1 [file ao5c12581_si_001.pdf]

Synthesis of NiRu-Layered Double Hydroxide for Enhanced Oxygen Evolution Reaction

Eden Argaw, Binod Raj KC, and Bishnu Prasad Bastakoti*

Department of chemistry, North Carolina A&T state university ,1061 E. Market St., Greensboro, NC 27411, USA.

E-mail: bpbastakoti@ncat.edu

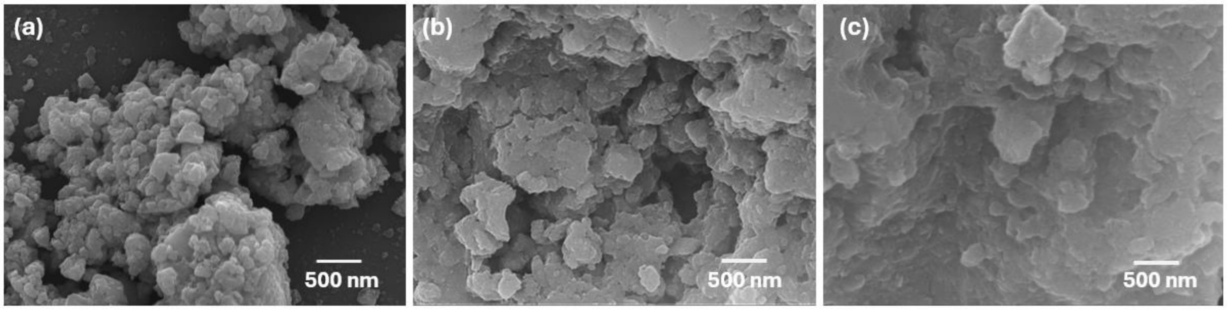

Figure S1: SEM images of (a) NiRu-LDH-20, (b) NiRu-LDH-50, and (c)NiRu-LDH-80 at a magnification of 500 nm.

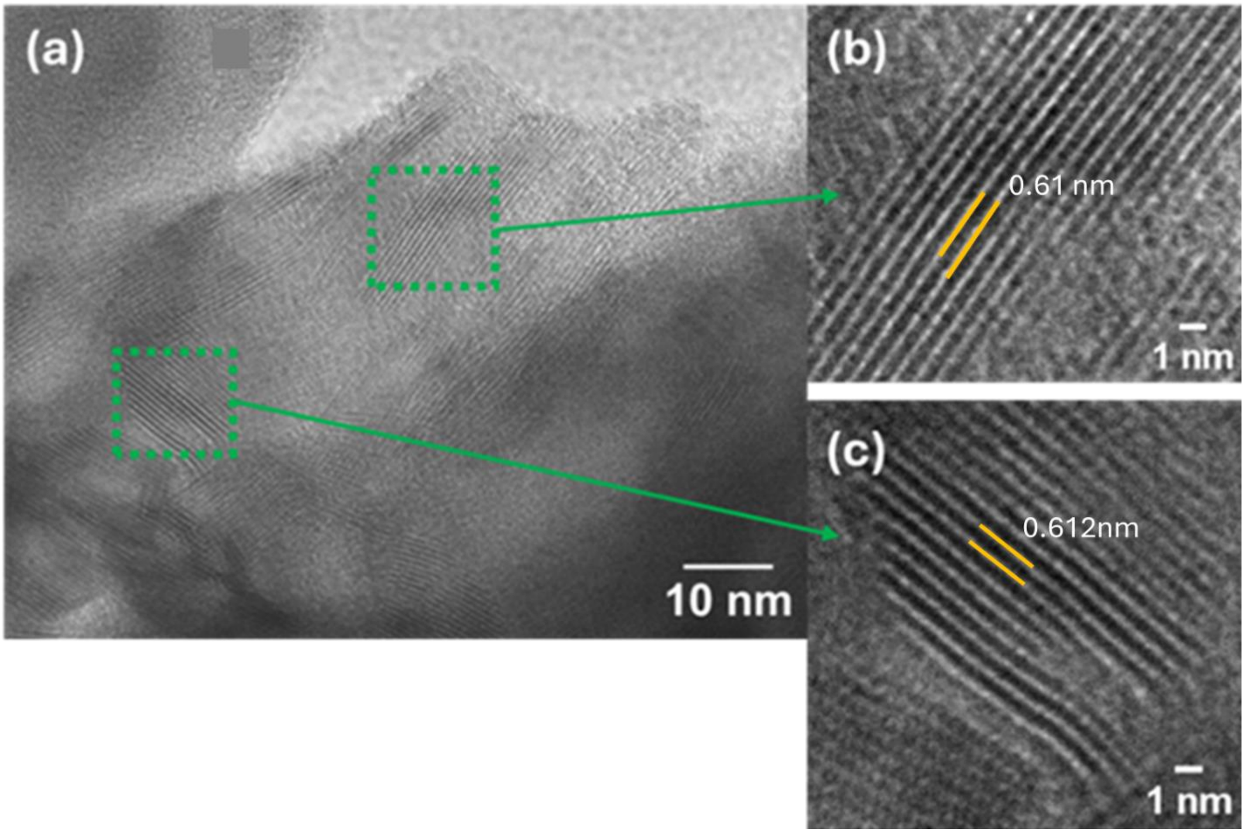

Figure S2: (a) High-resolution TEM image of NiRu-LDH showing clear lattice fringes corresponding to the (003) plane; (b) corresponding d-spacing measurement extracted from the lattice fringes; (c) lattice fringes of the (003) plane observed from a different selected region, confirming structural uniformity.

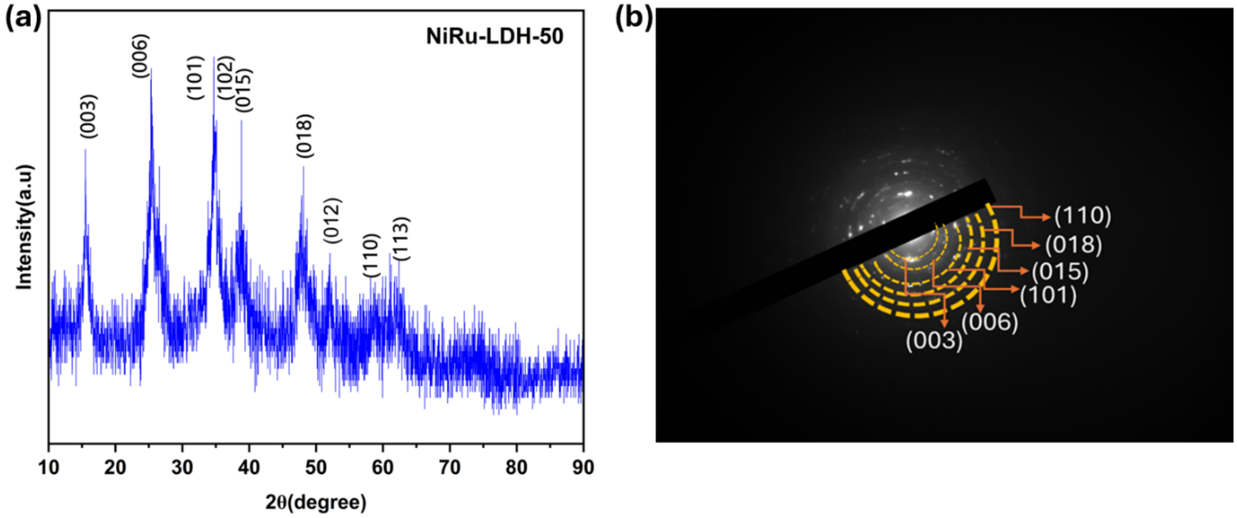

Figure S3: (a) XRD pattern of NiRu-LDH-50, (b) SAED pattern of NiRu-LDH-50 with indexed diffraction rings corresponding to the LDH phase.

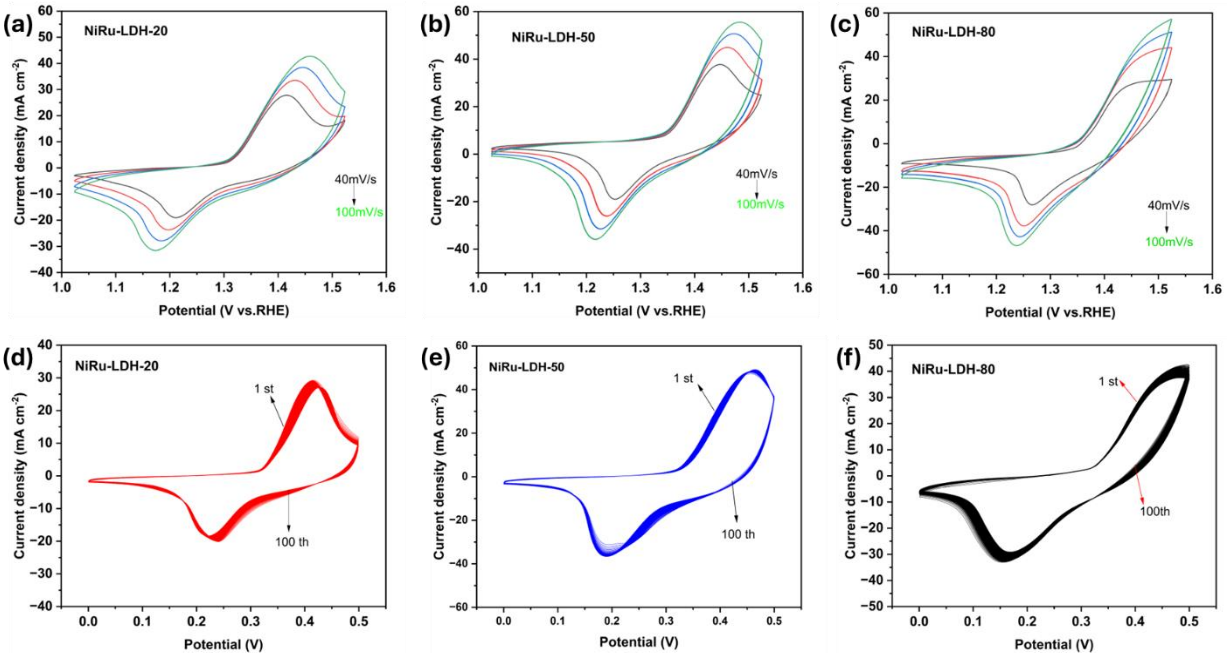

Figure S4: CV of (a) NiRu-LDH-20, (b)NiRu-LDH-50, (c) NiRu-LDH-80. 100 cycles CV of (d) NiRu-LDH-20, (e) NiRu-LDH-50, (f) NiRu-LDH-80 at 50mVs^{-1} scan rate.

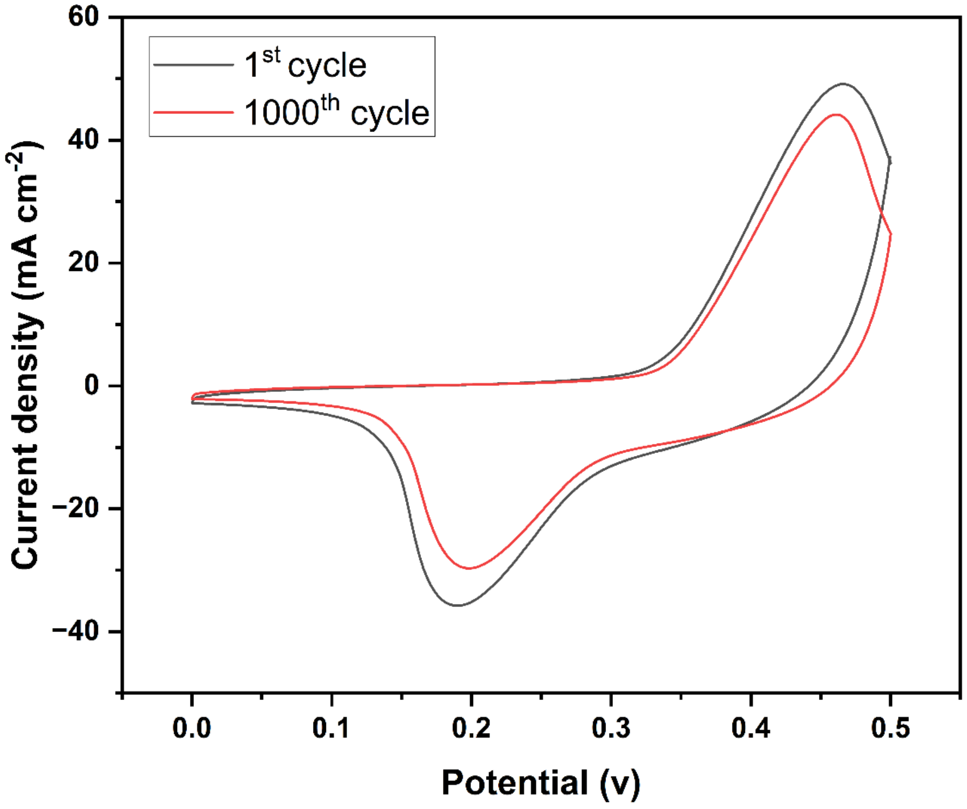

Figure S5: 1000 cycle CV of NiRu-LDH-50 at 50mVs^{-1} scan rate

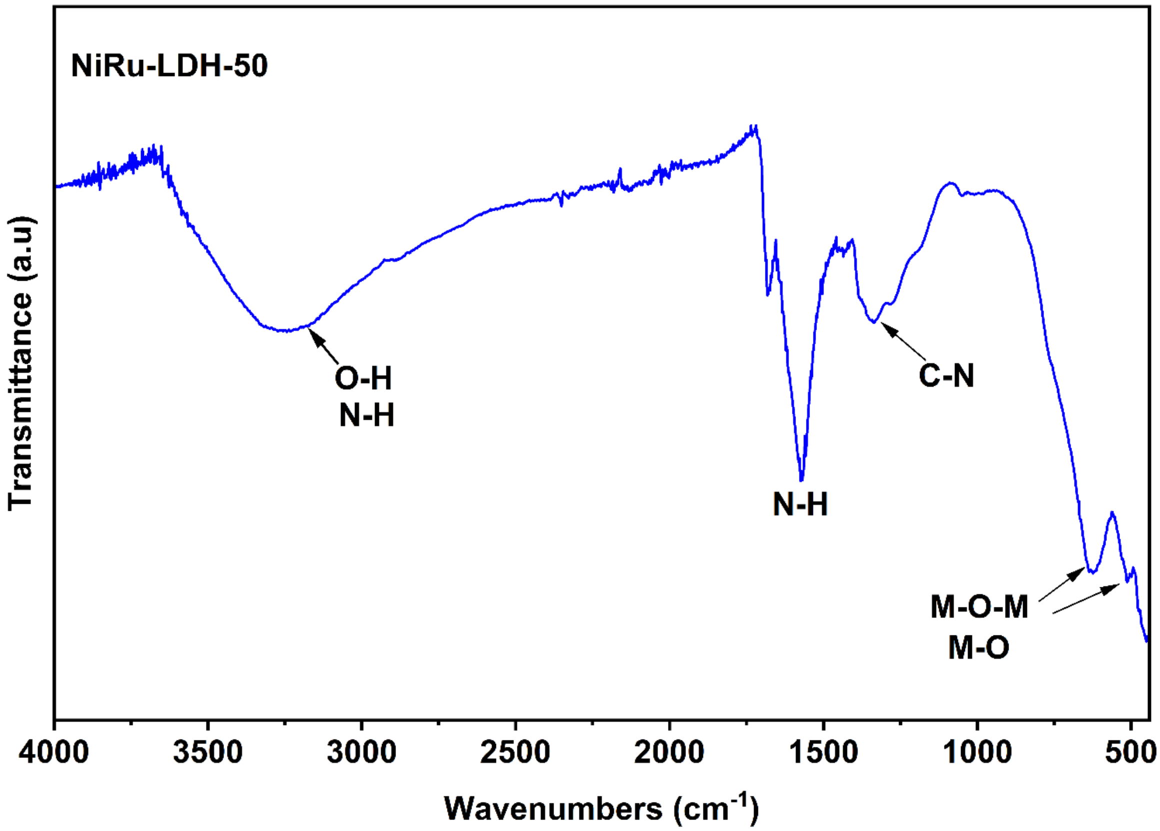

Figure S6: FTIR spectrum of the NiRu-LDH-50 sample.

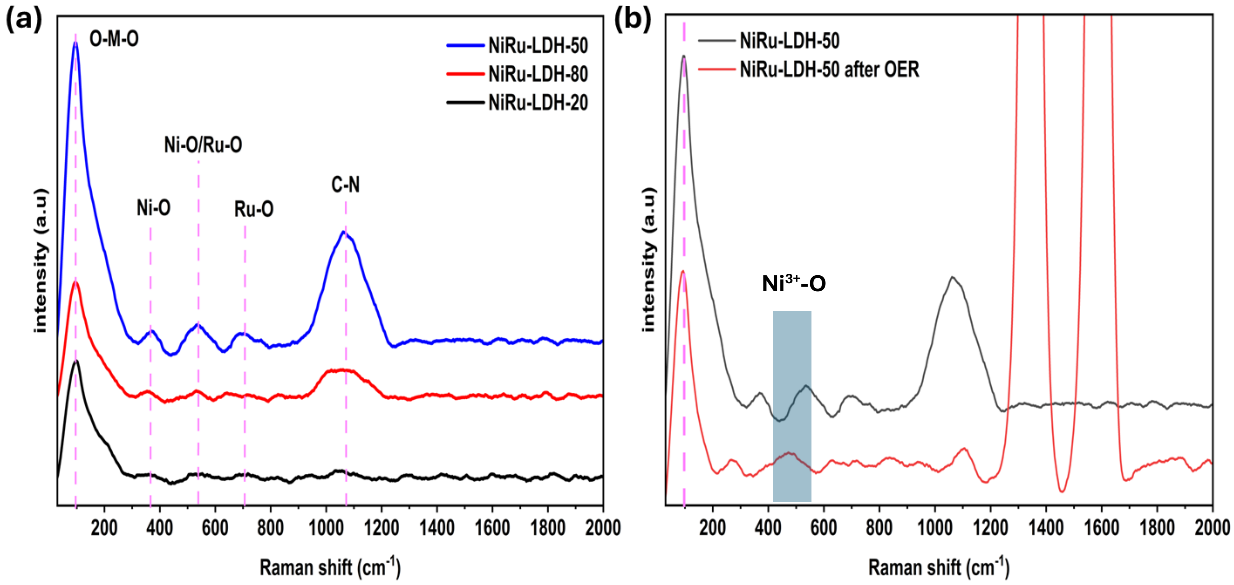

Figure S7: (a) Raman spectra of NiRu-LDH samples synthesized at different temperatures: NiRu-LDH-20, NiRu-LDH-50, and NiRu-LDH-80, (b) Raman spectra of NiRu-LDH-50 before and after OER.

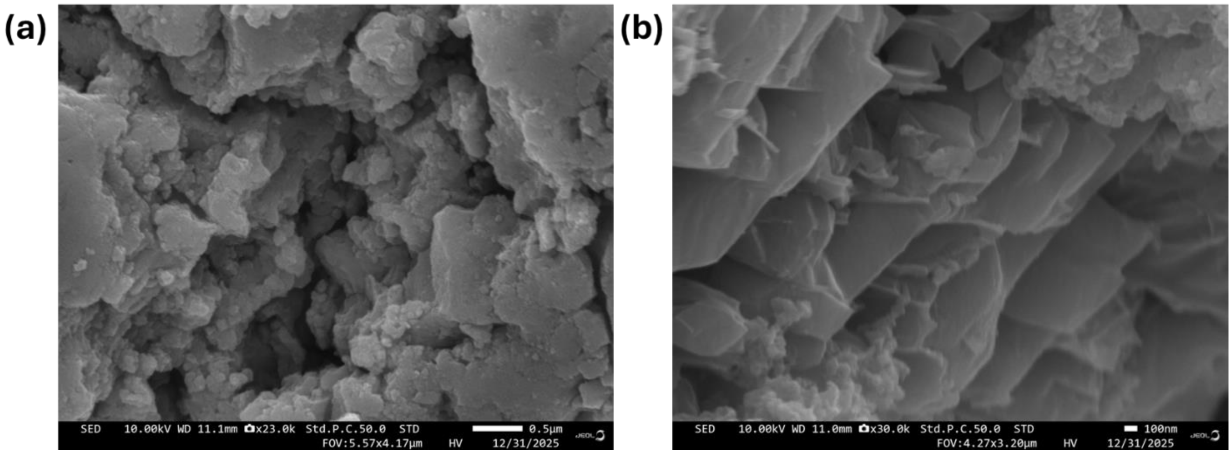

Figure S8: SEM images of NiRu-LDH-50 after OER testing at (a) 500 nm and (b) 100 nm magnifications.

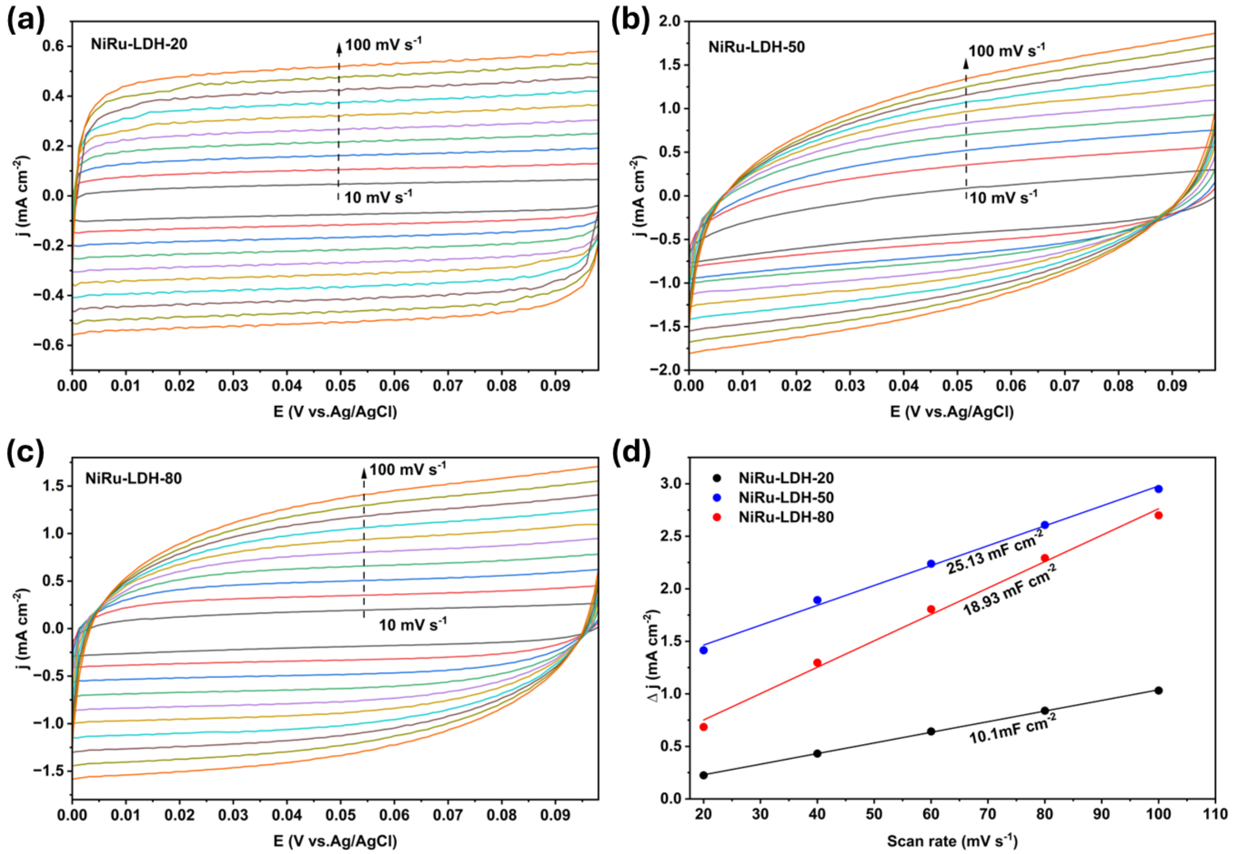

Figure S9: Cyclic voltammetry analysis in the non-faradaic region at different scan rates for electrochemical double-layer capacitance measurement of a) NiRu-LDH-20, b) NiRu-LDH-50, and c) NiRu-LDH-80, and d) corresponding plot of ΔJ versus scan rate for all samples.

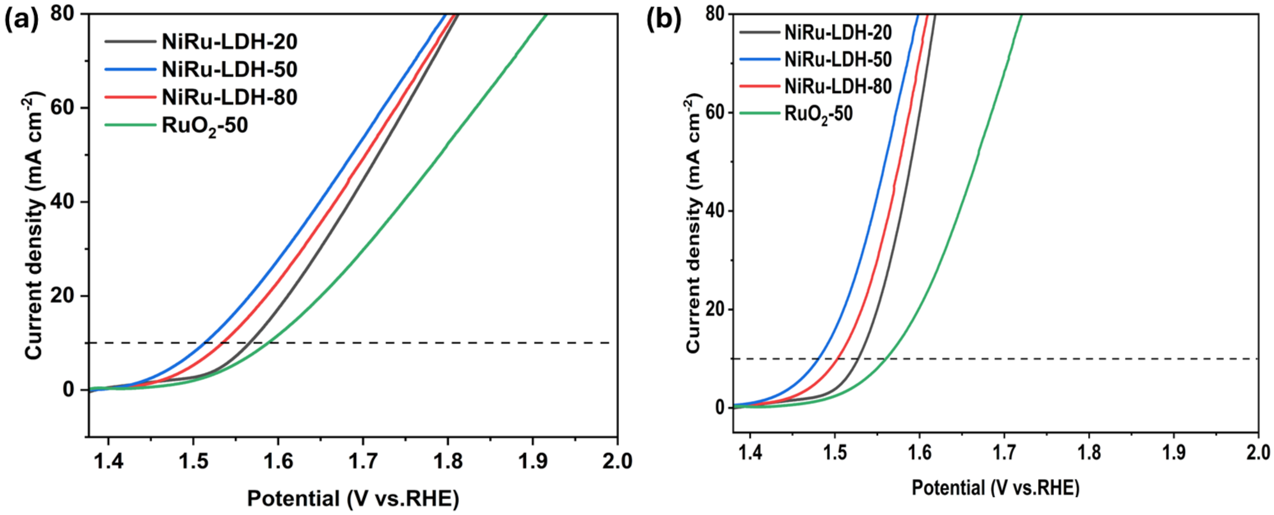

Figure S10: Linear sweep voltammetry (LSV) curves of all samples a) before *i*R correction and b) after *i*R correction in 1 M KOH.

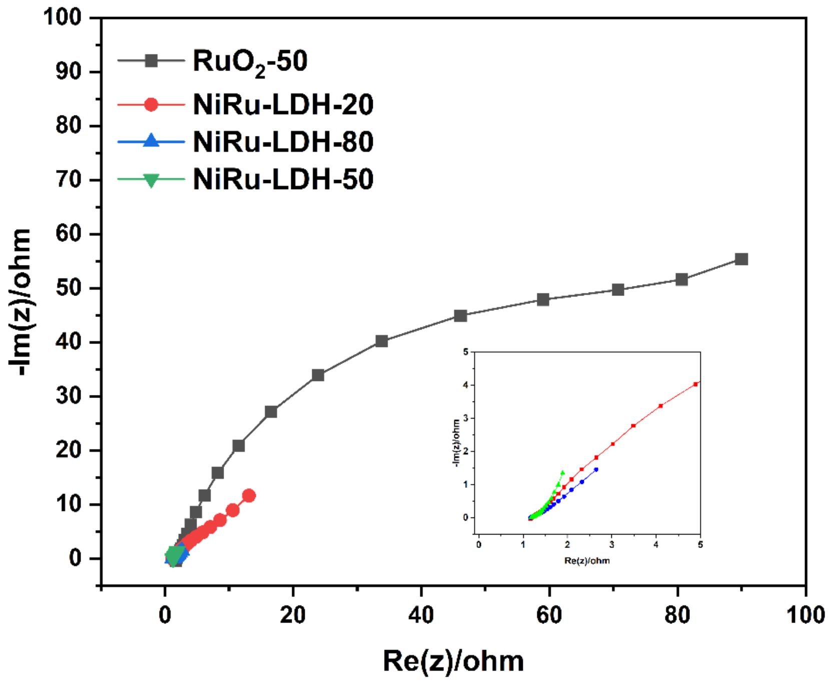

Figure S11: Electrochemical impedance spectra of NiRu-LDH at different synthesis temperatures and RuO_2 measured in 1 M KOH electrolyte with a frequency range of 100 kHz – 10 mHz.

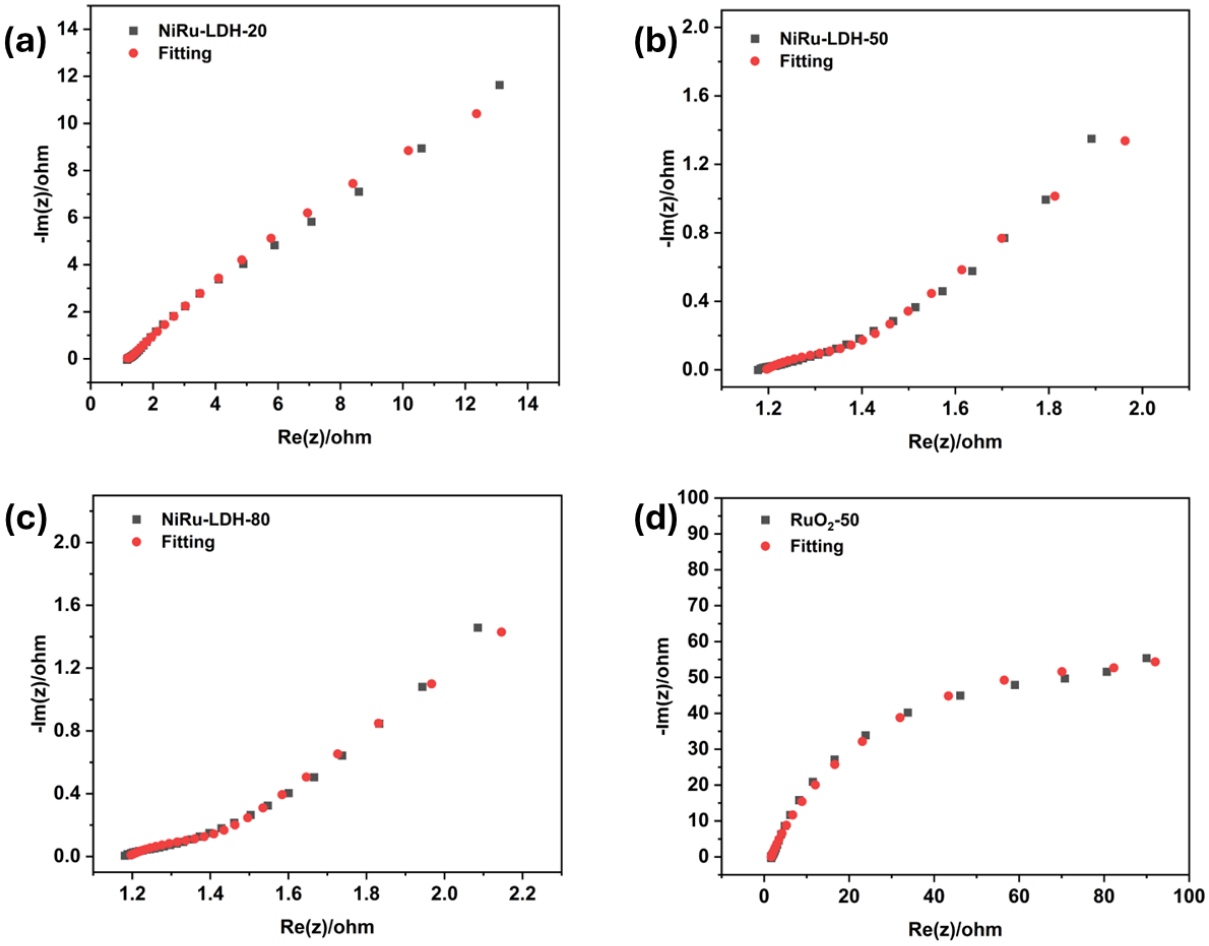

Figure S12. EIS measurements fitting data using Z-fi with frequency range of 100 kHz – 10 mHz in 1 M KOH, (a) NiRu-LDH-20, (b) NiRu-LDH-50, (c) NiRu-LDH-80, and (d) RuO_2-50

Electrochemically active surface area (ECSA) calculations

For ECSA determination, the difference in current density (ΔJ) between the anodic (J_a) and cathodic (Jc) currents was extracted at the midpoint of the selected non-faradaic potential window from CV curves recorded at various scan rates. The double-layer capacitance (Cdl) was obtained from the slope of the linear plot of ΔJ as a function of scan rate (ν), following the relation $C_{dl} = \frac{\Delta J}{\nu}$ $(Eq\ S1)$.[1] The ECSA of each sample was then estimated using ECSA $= \frac{C_{dl}}{C_S\ Eq}$ $(Eq\ S2)$, where $C_S$is the specific capacitance of a smooth surface, taken as 0.04 mF cm^{-2}.

The turnover frequency (TOF):- TOF was determined using the equation:

$$\text{TOF} = \frac{J \times A}{4 \times F \times n} \quad (Eq\ S3)$$

whereJ is the current density at the given overpotential (calculated at an overpotential of 300 mV) A is the electrode surface area, F is the Faraday constant (96485 C mol^{-1}), and n represents the moles of active material deposited on the electrode.

Supplementary Tables

Table S1: EIS data for the OER were fitted using the Armstrong and Henderson equivalent circuit

The equivalent circuit used to fit the EIS data	Name of sample	R_s (Ω)	R_{ct} (Ω)	R_p (Ω)	Cp (μF cm^{-2})
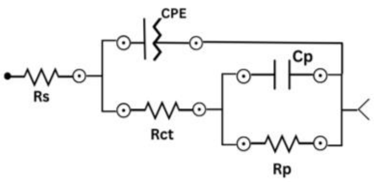	NiRu-LDH-20	1.199	7.419	97	249
	NiRu-LDH-50	1.2	1.16	16.6	204
	NiRu-LDH-80	1.411	2.575	24.2	158
	RuO_2-50	1.221	74.82	142	243

Table S2: Surface atomic percentage of elements in NiRu-LDH-50 determined from XPS analysis.

Name	Atomic Percentage
Ru 3p	5.34
O 1s	79.73
Ni 2p	14.93

Table S3: Electrochemical double layer capacitance (C_{dl}) and electrochemically active surface area (ECSA) for different electrocatalysts.

Samples	C_{dl} (mF)	ECSA (cm^2)
NiRu-LDH-20	5.05	126
NiRu-LDH-50	12.57	314
NiRu-LDH-80	9.47	237

Table S4: Comparison of OER overpotentials at 10 mA cm^{-2} in alkaline electrolyte

Catalysts	Methodology	Overpotential (mV) at 10 $mAcm^{-2}$	Electrolyte KOH	Reference
NiRu-LDHs	hydrothermal	380	0.1 M	[2]
Ag NP/NiRu-LDHs	hydrothermal	310	0.1 M	[2]
NiCo-LDH	hydrothermal	367	1 M	[3]
NiFe-LDH	hydrothermal	290	1M	[4]
NiCo LDH	solvothermal	290	0.1M	[5]
NiCo-LDH	sol–gel method and carbothermal reduction	330	1 M	[6]
NiRu-LDH	Coprecipitation	280	1 M	This work

References

(1) KC, B. R.; Yusa, S.; Bastakoti, B. P. Facile One-Pot Block Copolymer-Mediated Solvothermal Approach for Synthesis of High-Entropy Alloy with Enhanced OER Activity. *Precis. Chem.* **2025**, https://doi.org/10.1021/prechem.5c00094.

(2) Chala, S. A.; Tsai, M.-C.; Su, W.-N.; Ibrahim, K. B.; Duma, A. D.; Yeh, M.-H.; Wen, C.-Y.; Yu, C.-H.; Chan, T.-S.; Dai, H. Site Activity and Population Engineering of NiRu-Layered Double Hydroxide Nanosheets Decorated with Silver Nanoparticles for Oxygen Evolution and Reduction Reactions. *Acs Catal.* **2018**, *9*, 117–129.

(3) Liang, H.; Meng, F.; Cabán-Acevedo, M.; Li, L.; Forticaux, A.; Xiu, L.; Wang, Z.; Jin, S. Hydrothermal Continuous Flow Synthesis and Exfoliation of NiCo Layered Double Hydroxide Nanosheets for Enhanced Oxygen Evolution Catalysis. *Nano Lett.* **2015**, *15*, 1421–1427.

(4) Zhong, H.; Liu, T.; Zhang, S.; Li, D.; Tang, P.; Alonso-Vante, N.; Feng, Y. Template-Free Synthesis of Three-Dimensional NiFe-LDH Hollow Microsphere with Enhanced OER Performance in Alkaline Media. *J. energy Chem.* **2019**, *33*, 130–137.

(5) Jiang, J.; Zhang, A.; Li, L.; Ai, L. Nickel–Cobalt Layered Double Hydroxide Nanosheets as High-Performance Electrocatalyst for Oxygen Evolution Reaction. *J. Power Sources* **2015**, *278*, 445–451.

(6) Zheng, Y.; Bai, Y.; Lu, J.; Yuan, K.; Wu, X.; Cui, S.; Shen, X.; Ma, X.; Zhu, J. Constructing of Non-Precious Metal-Based NiCo Bimetallic Aerogel by Thermal Reduction of Carbon Species under Elevated Temperatures for Efficient Oxygen Evolution Reaction. *J. Mater. Sci. Technol.* **2025,** *242*, 52-63.
